# Non-Motor Aspects of Tic Disorders—New Developments

**DOI:** 10.3389/fpsyt.2016.00213

**Published:** 2017-01-09

**Authors:** Daphna Ruhrman, Ella Gev, Noa Benaroya-Milshtein, Silvana Fennig, Orit Krispin, Alan Apter, Tamar Steinberg

**Affiliations:** ^1^The Matta and Harry Freund Neuropsychiatric Tourette Clinic, Schneider Children’s Medical Center of Israel, Petach Tikva, Israel; ^2^Sackler Faculty of Medicine, Tel Aviv University, Tel Aviv, Israel

**Keywords:** tic disorders, premonitory urge, acceptance, sensory modulation, environmental influences, attachment, emotion dysregulation

## Abstract

The cardinal characteristics of tic-related disorders are stereotyped motor movements and vocalizations. However, they may be accompanied by non-motor features that appear sequentially during the course of the disorder and can sometimes be more disabling than the tics themselves. This review presents our perspectives on several non-motor aspects of Tourette syndrome based on the long experience of the Neuropsychiatric Tourette Clinic of a tertiary pediatric medical center. The effect of premonitory urges, sensory modulation disorder, tic-related cognitions, and environmental conditions on the expression and intensity of tics is elaborated, with suggestions for treatment approaches to each. We also describe the mediatory effect of parental attachment style on the link between maternal stress and ticcing intensity and the need to adjust psychotherapy interventions to account for the importance of this factor in emotion regulation. This review is intended to direct attention to the non-motor aspects of Tourette syndrome. An in-depth understanding of this complex and debilitating disorder will facilitate the formulation of innovative therapeutic protocols.

## Background

The stereotyped motor movements and vocalizations that are the cardinal characteristics of tic disorders are often accompanied by non-motor features that appear sequentially during the course of the disorder and can sometimes be more disabling than the tics themselves. The Neuropsychiatric Tourette Clinic of Schneider Children’s Medical Center of Israel, a tertiary university-affiliated pediatric hospital, has long experience in treating children with Tourette syndrome and its associated clinical manifestations. The aim of this review is to share our subjective perspectives on the non-motor aspects of Tourette syndrome based on findings and observations in our clinic, as presented at the first World Congress on Tourette Syndrome and Tic Disorders (1). We start with premonitory urges, which play a significant role in tic expression and tic sequence, and the impact of acceptance procedures on decreasing their intensity. This is followed by a discussion of sensory modulation disorder (SMD) as an important component of the reaction to premonitory urges and as a comorbidity of Tourette syndrome, in addition to the development of tic-related cognitions and their influence on tics and tic-related behaviors. The effect of environmental factors on tic expression and their bearing on the development and refinement of behavioral treatment strategies are elaborated. There is also a section on the relevance of attachment theory and the modulatory role of parental attachment style in the relationship between children with Tourette syndrome and their parents.

## Premonitory Urges

Studies have shown that premonitory urges play a significant role in tic expression (2–6). However, the exact functional relationship between premonitory urges and tics has not been explored.

A premonitory urge is an internal physical sensation that is experienced as a drive to perform a movement, either motor or vocal. Specifically, it is an impulse to tic. In a cross-sectional study of 135 patients aged 8–71 years with tic disorder, Leckman et al. (2) found a 95% prevalence rate of premonitory urges. The exact experience of premonitory urges, however, is extremely diverse. They have been described as an itch, a burn, an energy that must be released, a need to release tension, and a mental will (7). Some individuals fail to discriminate the physical experience of premonitory urges from the tics themselves (5, 7). Studies have repeatedly shown that the premonitory urges and the internal struggle to control them may be even more debilitating than the tics themselves. We have found that psychoeducation may help patients distinguish premonitory urges and reduce their discomfort.

The mean age at which patients with Tourette syndrome first become aware of premonitory urges is 10 years, an average 3.1 years after onset of the tics (2). Our clinical experience has shown that younger children (<10 years) can identify premonitory urges, but older children report them more clearly and consistently (8). Furthermore, urge ratings correlate with tic severity only in older children. These findings may be attributable to the increase in cognitive maturity with age (3, 5).

Several instruments have been developed to help children capture the urge experience using various definitions and descriptions. The well-established Premonitory Urge Scale for Tics (PUTS) (5) was found to facilitate premonitory urge recognition in children as young as 8 years and is widely used to measure the intensity of urges. In addition, therapeutic programs such as exposure and response prevention (ERP), habit reversal therapy (HRT), and comprehensive behavioral intervention for tics (CBIT) can initially assist young children in verbally describing what they feel during tics. Using these tools or similar ones, researchers found that urge frequency and intensity decreased in response to medications such as neuroleptics and relaxation and concentration exercises and increased in states of stress and anxiety (2). Others, including a group from our center, reported that the intensity of premonitory urges increased during tic suppression (6, 9), suggesting that the urges may serve as precipitators of tics (10) and that tic performance is directed at alleviating the premonitory urges (3). This was supported by studies using ERP protocols (11–13). Himle et al. (6) postulated that urges are an aversive experience and tics serve as a response to them and are negatively reinforced by urge removal. Accordingly, tic suppression temporarily increases the salience of the urge. Thus, when a child is allowed to tic freely, the premonitory urge is relatively low in intensity (6). However, whether premonitory urges are a conditioned stimulus for tics remains controversial, as other studies failed to find changes in self-reports of urge intensity during tic suppression (14, 15). As in most instances of mind–body relations, the urge–tic relationship is not linear and clear-cut. Subjects with tics may adopt the belief that the tics are the best solution for the unease caused by premonitory urges. The tics themselves later become aversive themselves because they do not allow the individual to relax or concentrate, and in severe cases, may even result in serious injury. This situation is exacerbated when the environment responds negatively to the tics. Accordingly, Capriotti et al. (16) found that the intensity of the premonitory urge is correlated with the aversive consequences of ticcing. Therefore, clinicians need to address both components of the premonitory urge–tic complex and help children find the most suitable way to deal with each. Our group uses the acceptance-based approach of the acceptance and commitment therapy (ACT) protocol proposed by Hayes and Wilson (17). These researches suggested that attempts to control aversive sensations are not only ineffective, they may even amplify the experience the individual was trying to avoid. However, accepting these experiences, that is, viewing them as only something to be experienced reduces their negative impact (17, 18). To test this hypothesis, we compared urge intensity, frequency, and discomfort with tic frequency under three conditions: ticcing freely, tic suppression, and urge acceptance (using mindfulness techniques and diaphragmatic breathing). The results showed that the frequency and intensity of the urges was significantly reduced in the urge acceptance condition compared to the others. A similar pattern was found for the level of discomfort caused by urges. We also noted a significant reduction in tic expression in the urge acceptance condition compared to the freely ticcing condition, suggesting that accepting urges can help children not only to cope with premonitory urges but also to reduce tic expression (9). This finding was supported by a recent study by Reese et al. (19) showing a correlation between a reduction in tic severity and increased mindfulness. Thus, premonitory urges may be considered a core aspect of Tourette syndrome, and they need to be distinguished from the tics in order to improve our understanding and treatment of the disorder as a whole. The experience of our group suggests that alternative approaches of acceptance aimed specifically at premonitory urges should be incorporated into modern treatment protocols for tics.

## SMD

Sensory modulation disorder is an impairment in regulation of the degree, intensity, and nature of responses to sensory input (either over- or under-sensitivity), with an adverse effect on activities and routines of daily living (20). A study from our clinic showed that the prevalence of SMD is considerably higher in children with Tourette syndrome (34.7%) than in healthy children (<5%) (21), suggesting that SMD is a comorbidity of Tourette syndrome. The presence of SMD can directly affect how individuals with Tourette syndrome respond to premonitory urges. That is, if the premonitory urge constitutes an over-reactivity to sensory input (before emergence of the tic), and SMD creates an over-attentiveness to aversive sensory input, then children with Tourette syndrome may feel obligated to tic to rid themselves of the unpleasant inner stimulus.

Children with both SMD and Tourette syndrome also have high rates of other common comorbidities, such as obsessive–compulsive disorder (OCD), depression, and attention-deficit and hyperactivity disorder (ADHD), which significantly impact their quality of life. We speculate that children with SMD and Tourette syndrome undergo a long learning process, beginning at a very young age and continuing toward adulthood, during which they incorporate the intense relationship between sensory inputs and the immediate response to them. As a consequence, a strong link evolves between the two, creating the illusion that they constitute a single entity. The challenge during treatment is to break this link (21).

## Tic-Related Cognitions

Studies of illness-related beliefs have shown that the beliefs and expectations (cognitions) of patients about their illness or somatic symptoms play an important role in the impact of the illness on their life. In individuals with Tourette syndrome, tic-related cognitions develop in childhood along with the experience of tics and premonitory urges. The cognitions include appraisals and beliefs regarding inner (premonitory urges) and outer (environmental) sensory inputs, responses to these inputs (tics), and the ability to express, suppress, or modify one’s responses. To investigate the different thoughts of children about the origin and consequences of their tics, we designed a self-report inventory, the Beliefs About Tic Scale (BATS) (22), which we administered to a sample of 56 patients aged 10–18 years with Tourette syndrome. The results showed that patients’ negative beliefs about their ability to suppress tics (or to resist premonitory urges) were related to higher perceived urge intensity (measured by PUTS scores). Apparently, the perception of a lack of control increases the adverse emotional consequences of premonitory urges. This illness-related distress can be triggered by cognitive as well as emotional processes (23). Our findings are in line with the study of tinnitus by Sirois et al. (24) showing that among patients with the same symptom severity, those who believed they had control over their condition had lower levels of depression and a better quality of life. Additionally, our group showed that the level of depression was highly correlated with negative beliefs about the ability to suppress (i.e., control) tics, but only in children older than 13 years (22). We also noted several positive correlations between perceived urge intensity (measured by PUTS scores) and psychiatric symptoms. In children with OCD, PUTS scores were correlated with OCD severity, again only in those older than 13 years. There was a correlation between PUTS scores and obsessions, which increased with an increase in age, to almost complete congruence (22). Hence, premonitory urges can be considered a type of “obsession” with bodily sensations. These findings are in agreement with the suggestion of O’Connor (25) that children with Tourette syndrome have excess sensitivity to sensory stimuli and as a result tend to associate negative feelings with perceptions. This in turn leads to exaggerated negative perceptual biases, comparable to the cognitive distortions found in depression and anxiety. It is probable that as the child grows, he/she learns to associate these negative appraisals with the urges and tics. The negativity becomes increasingly more debilitating and bothersome and may take the form of an obsession. The lack of control associated with tics accentuates these negative feelings, and quality of life is diminished (25).

## Environmental Factors

Although tics arise from a disturbance in the underlying brain circuitry, there is increasing evidence that tic expression may be either exacerbated and attenuated by environmental and psychological factors (13), such as psychosocial stress (26). In their literature review, Conelea and Woods (27) identified a variety of situations associated with changes in tic frequency, including boredom and passive states, social gatherings, concentration on a task, watching TV, and playing sports. The consequences of tic expression may also influence its appearance. These may be external, such as social attention (teasing, comforting, release from demanding tasks) or internal (subjective feeling of relief of a premonitory urge after tick, shame, or guilt) (28, 29). Studies of the impact of environmental factors on tic expression have improved our understanding of the etiology and maintenance of tics. They have also influenced the strategies used to treat tic disorders. Behavioral treatment approaches, such as HRT, are based on the rationale that despite their biological origin, tics may be worsened, improved, or maintained by environmental events. Treatment is therefore partly directed at systematically identifying and modifying events or experiences that contribute to tic severity (30).

The mechanisms whereby different environmental conditions affect tics have been hardly investigated, apart from those related to learning theories and emotional effects. Therefore, we conducted a study at our clinic in which tic expression was evaluated under five challenging environmental situations: watching television; doing homework; being alone; receiving attention when ticcing; and talking to a stranger study (1). Two measures were used: a subjective measure consisting of a structured interview in which children were asked to describe the level of tics in these situations, and an objective measure consisting of a video recording of the child’s response in each situation. The results yielded differential effects of the different environmental situations on tic expression. The objective measure revealed that the highest number of tics appeared in the watching television situation, and the lowest, in the alone situation. Combined with the report on the effect of stress on tic severity by Conelea et al. (31), our findings suggest that highly stimulating environmental conditions can interfere with motor inhibition and thereby exacerbate tic expression. Therefore, the higher the level of stimulation in each of our situations, the greater the number of tics expressed. By contrast to the objective measure, the subjective self-reports of the subjects served only as a moderate–low predictor of the effect of the environment on tics. This points to a low level of patient self-awareness of the impact of the environment on the performance and intensity of tics. It may also indicate a low reliability of subjective reports of tic severity, which form the basis of most clinical and research evaluations. Interestingly, the subjective reports were more in line with the objective measure in the presence of strong premonitory urges. Thus, it is possible that the tic brings the urge to the child’s attention, increasing his/her awareness of both the tic and the environmental influences. We suggest that alerting children to environmental factors that affect tic performance may help them acquire better coping skills in situations associated with tic exacerbation.

## Parent–Child Attachment

The parent–child relationship is a basic component of the clinical understanding of children with Tourette syndrome. Our group attempted to investigate this aspect of Tourette syndrome through the prism of attachment theory (32), which has been found to serve as a useful framework for understanding parent–child relationships under conditions of stress (33, 34). As noted above, the psychological dynamic of children with Tourette syndrome is often complicated by comorbidities involving impaired emotion regulation, such as OCD, ADHD, anxiety disorders, depression, and rage attacks (35), which can be a major impediment to normal childhood development (35, 36). We focused on the reciprocal effect of emotion dysregulation in a developmental context and the experience of self between children with Tourette syndrome and their parents and the impact of these interactions on the child’s mental health (32). Parents of children with Tourette syndrome are subject to a vicious cycle on a daily basis of the child’s emotion dysregulation on the one hand and their own inability to serve as a container on the other. This cycle is dictated by two interrelated factors: the characteristics of the parent–child relationship and the child’s self-representation and emotion-regulation abilities. Insights for the study were derived from Bowlby’s attachment theory (37), which claims that children tend to seek the protection of the parent or significant other in order to gain security. To survive, they form an array of internal conscious and unconscious representations of the self and related others, and these determine their attachment style: secure or insecure (anxious and/or avoidant). Children with a secure attachment style have internal working models of others as positive and protective. In times of need, they search for comfort and support from the other in order to achieve emotion regulation, a cornerstone of good mental health. Children with an anxious or avoidant attachment style have representations of others as unavailable, rejecting, or even harming, and acquire a lasting fear of rejection and abandonment. Thus, their ability to get comfort and help from others is damaged. The broadening of the theory to apply to adulthood, suggesting that the system that gives rise to the emotional bond between children and parents continues to play a role when the children reach adulthood and confront obstacles of parenting (33, 38). Our study focused on the relationship between parental stress and attachment style and its influence on tic severity and impairment (32). We showed that an increase in the level of maternal stress, as measured by the mother’s perception of the child as “difficult” using a subscale of the Parental Stress Index (39), was associated with an increase in the intensity of the child’s ticcing. These findings pave the way for designing new clinical interventions for both children with Tourette syndrome and their parents based on the attachment theory. Vulnerable families should be identified by their attachment style, followed by adjustments to the psychotherapy intervention, mainly with a focus on the parents, in order to enhance their ability to identify, adjust to, and fulfill the child’s needs in terms of emotion regulation. In this manner, common comorbidities of Tourette syndrome can be better managed and children will derive greater benefit from existing tic-targeted treatments, ensuring their healthier mental development.

## Conclusion

Tourette syndrome is a complex disorder, comprising both motor and non-motor/emotional components. This review was intended to direct attention to the non-motor aspects of Tourette syndrome and to encourage further research into their role in the underlying mechanism of the disorder. An in-depth understanding of this disabling condition will lead to better and more innovative treatments.

## Author Contributions

All the authors listed have made substantial, direct, and intellectual contribution to the work and approved it for publication.

## Conflict of Interest Statement

The authors declare that the research was conducted in the absence of any commercial or financial relationships that could be construed as a potential conflict of interest.
